# Variation in the oxytocin receptor gene moderates the protective effects of a family‐based prevention program on telomere length

**DOI:** 10.1002/brb3.423

**Published:** 2016-01-17

**Authors:** Erica L. Smearman, Tianyi Yu, Gene H. Brody

**Affiliations:** ^1^ Behavioral Sciences and Health EducationRollins School of Public HealthEmory University1518 Clifton Road NortheastAtlantaGeorgia30322; ^2^ Center for Translational and Social NeuroscienceEmory UniversityAtlantaGeorgia30322; ^3^ Center for Family ResearchUniversity of Georgia1095 College Station RoadAthensGeorgia30602‐4527

**Keywords:** Gene–environment interaction, intervention, OXTR, oxytocin, parenting, telomere

## Abstract

**Introduction:**

Parent–child relationships with high conflict and low warmth and support are associated with later adverse behavioral and physiological child outcomes. These outcomes include shorter telomere lengths, the repetitive sequences at the ends of chromosomes that have been utilized as a biomarker for chronic stress. Our research group furthered this by exploring telomere length outcomes following a family‐based prevention program and identified reduced telomere shortening 5 years post intervention among those originally exposed to nonsupportive parenting and randomized to the intervention condition. However, not all individuals respond equally, and a growing literature suggests genetic sensitivity to one's environment, with variations in the oxytocin receptor gene (*OXTR*) potentially influencing this sensitivity.

**Methods:**

We utilized data from African American youths (mean age 17) randomized to intervention (*n* = 100) or control condition (*n* = 91) with baseline assessments of genetic status and nonsupportive parenting, and 5‐year follow‐up assessments of telomere length.

**Results:**

We found a significant three‐way interaction between nonsupportive parenting, intervention condition, and *OXTR* rs53576 genotype. *OXTR* GG individuals, who are suggested to be more sensitive to their social environment, exhibited significantly more variability, evidencing the shortest telomeres when exposed to nonsupportive parenting and randomized to the control condition, and similar telomere lengths to non at‐risk groups when randomized to the intervention. In contrast, those with the A allele showed no statistical difference in telomere lengths across parental and intervention conditions. Subsequent analyses suggest that these findings may be mediated through chronic anger, whereby GG individuals exposed to nonsupportive parenting and randomized to the control condition had a greater increase in chronic anger by study follow‐up, compared to those in the intervention, and this change associated with greater telomere shortening.

**Conclusions:**

These findings highlight the importance of individual differences and potential role of genetic status in moderating the relationship between environmental contexts and biological outcomes.

## Introduction

Exposure to chronic stressors such as parent–child relationships with high levels of conflict and low levels of warmth and emotional support have repeatedly been associated with later adverse behavioral and physiological outcomes (Repetti et al. 2002; Miller et al. 2011). Indeed, this link between exposure to chronic stressors in youth and later poor outcomes has become well‐established and has resulted in a call for increased attention to public policy and interventions designed to reduce exposure to these conditions or their effects (Shonkoff 2010). However, questions remain about how to best measure the impact of such interventions, especially given that outcomes of interest such as improved health and behavior can occur a number of years in the future. Telomeres have emerged as a possible option for addressing this question.

Telomeres are repetitive sequences on the ends of chromosomes that serve as protective caps. During cell division, the ends of the chromosome are not replicated resulting in shorter telomeres following cell division (Blackburn 2005), though telomere length can be increased by the enzyme telomerase, allowing for some dynamic regulation (Blackburn 2005). Interestingly, in addition to age, telomere shortening has repeatedly been reported under conditions of chronic psychosocial stress (Epel et al. 2004), including stress that occurs during childhood (Cohen et al. 2013; Price et al. 2013; Shalev et al. 2013; Mitchell et al. 2014). Furthermore, telomere shortening has also been predictive of future poor health (Epel et al. 2006; Fitzpatrick et al. 2007; Cohen et al. 2013) and mortality (Cawthon et al. 2003; Bakaysa et al. 2007). While the mechanisms by which this shortening occurs are still being explored, including the potential for increased cell replication or weakened telomerase activity following activation of stress response systems (Choi et al. 2008; Epel et al. 2010; Tomiyama et al. 2012), investigating telomere length in the context of stress and long‐term behavioral and health outcomes may have implications for intervention research. If intervention programs can result in reduced telomere shortening, this may suggest a positive impact of interventions at a biological level and may forecast a greater likelihood of improved mortality and future health.

Recent pilot studies suggest that preventative interventions may have protective effects on telomere length (Epel et al. 2009; Jacobs et al. 2011; Daubenmier et al. 2012) and spurred the incorporation of telomere length assessment in the Adults in the Making (AIM) prevention program (Brody et al. 2010, 2012). AIM is an efficacious family‐centered, skill‐based prevention program for African American adolescents in the rural south primarily aimed at building family stress‐buffering processes. At age 17, when youth were initially randomized to the 6‐week intervention or control conditions, primary caregivers provided data on parental support including degree of conflict and level of warmth and emotional support provided to the youth. At age 22, 5 years after the intervention, telomere length was indexed from youth peripheral blood mononucleocytes.

Among youth exposed to high conflict and low warmth and emotional support at baseline, participation in the AIM intervention program impacted telomere length across 5 years. Exposure to these environments was associated with significantly shorter telomere length at the 5‐year follow‐up; however, this effect was found only for those randomized to the control condition (Brody et al. 2015). Participation in the intervention appeared to attenuate this relationship, with those in the intervention evidencing similar telomere lengths to the non at‐risk group. Subsequent analyses suggested that this may be through the intervention's influence on chronic anger. Chronic anger is reported as a common consequence of exposure to nonsupportive parenting (Brody et al. 2010; Simons et al. 2011), and has been linked with activation of stress response systems suggested to influence telomere shortening (Blackburn 2005; Aviv 2008). Those in the study's control group were found to have a greater increase in chronic anger ratings over 5 years, compared to those in the intervention, and these changes were then associated with telomere length (Brody et al. 2015).

The intervention's protective findings suggests that participation in efficacious intervention programs may have the potential to intervene on stress processes at a biological level and promotes the role of intervention programs among youth living in high conflict or stressful environments. While prevention programs may benefit all youth to a degree, there is a growing appreciation and literature on individual differences in sensitivity to one's environment, both positive and negative (Belsky and Pluess 2009). These differences may be partly explained by genetic predispositions. Certain genes, especially those involved in neurotransmission, may influence one's sensitivity to environmental contexts, with individuals evidencing greater behavioral and phenotypic outcomes following environmental exposures (Belsky et al. 2007; Belsky and Pluess 2009). Conditions with both negative and positive environments may provide a unique opportunity to explore these questions of genetic sensitivity.

Within the telomere literature, the hypothesis of genetic sensitivity was recently applied with a report showing that exposure to disadvantaged environments was associated with reduced telomere length at age 9, but that this association was moderated by genotype (Mitchell et al. 2014). Children with certain serotonin and, to a lesser degree, dopamine genotypes were reported to have the shortest telomeres when exposed to disadvantaged environments, compared to those without these genotypes. Studies have also applied this hypothesis to prevention programs, finding that genetics may influence the association between intervention participation and the degree of behavioral and phenotypic outcomes (Rutter 2005; Brody et al. 2014; Sales et al. 2014), yet no study to date has explored whether genetics may moderate the impact of prevention programs on telomere length outcomes.

Although a number of genotypes have been included in the sensitivity literature, due to the social nature of parent–child relationships and family‐based intervention programs, genetic variation in the oxytonergic system, a system important for bonding and perception of social cues (Bartz et al. 2011; Olff et al. 2013), may be particularly relevant when studying the impact of these environmental contexts. One of the most commonly studied polymorphisms in the oxytocin receptor gene (*OXTR*) is rs53576, which results from an adenine (A)/guanine (G) transition in the 3^rd^ intron. Interestingly, while the G allele was initially associated with more positive prosocial outcomes, such as trust (Krueger et al. 2012) and empathy (Rodrigues et al. 2009), in negative social contexts, individuals with the G allele have been found to display more adverse outcomes, such as greater emotional dysfunction (Bradley et al. 2011), decreased maternal sensitivity (Sturge‐Apple et al. 2012), and increased antisocial behaviors (Smearman et al. 2015). Furthermore, those with the G allele, compared to the AA genotype, were found to display significantly higher levels of resilience and positive affect when raised in high stability and warmth environments, yet significantly lower levels of resilience and affect when raised in low stability and low warmth environments (Bradley et al. 2013). These findings support the concept of genetic sensitivity and more specifically, the Social Sensitivity hypothesis for oxytocin, where individuals with the rs53576 G allele are thought to be more perceptive of social cues and thus more sensitive to, and responsive toward, the social environment (Bartz et al. 2011; Olff et al. 2013). In this regard, individuals with the G allele may be more impacted by nonsupportive family environments, yet may also be more responsive to family‐based prevention interventions.

Our study sought to expand upon this literature and the initial AIM study finding by evaluating the role of *OXTR* genetic status on the effect of intervention participation on later telomere length. A unique benefit of intervention programs is the ability to directly modify the environment in a controlled setting. This control allows for more direct testing of the association between environmental exposure and phenotypic outcomes and the ability to test for moderators of this association (Rutter 2005; Brody et al. 2013a, 2014). Therefore, our study proposed a three‐way interaction incorporating nonsupportive parenting, intervention condition, and *OXTR* genetic status on 5‐year follow‐up telomere lengths among AIM study participants. Aligned with the Social Sensitivity hypothesis for oxytocin, we hypothesized that individuals with the *OXTR* GG genotype would evidence the shortest telomeres when exposed to high levels of nonsupportive parenting and the control intervention condition, and longer telomere lengths when randomized to the intervention, while individuals with the A allele would be less impacted, evidencing relatively similar telomere lengths across all conditions. Furthermore, to follow the prior study, we subsequently tested whether changes in chronic anger may serve as a mediator between intervention participation and telomere length outcomes.

## Methods

### Participants

Participants were drawn from 367 African American youth who participated in the Adults in the Making (AIM) trial. Participants were in high school at the start of the study, 63.6% lived in single‐mother‐headed households, and 56.1% of participants were female. The majority of the youths' caregivers (78.7%) completed high school or earned a GED and median family income was $2012 per month, which can be described as working poor and is representative of the sampled population (Boatright 2005). Of the 367 who participated, 216 agreed to telomere length assessment at age 22 and a total of 191 had both telomere and genetic data. Using two‐factor multivariate analyses, there were no differences between the original and final samples on any demographic or study variables other than gender; the final sample had a lower percentage of females (*P *< 0.05). Gender was controlled for in all analyses. Of those in the final sample, 100 were randomized to the intervention condition and 91 to the control condition. Caregivers provided informed consent for their own participation and the participation of minor youth and youth provided assent, if under 18, or informed consent for participation. Families were compensated $100 at each assessment.

### AIM intervention

The AIM intervention program is modeled after an existing family‐based, skills‐training intervention designed to mitigate the negative impact of life stress on rural African American adolescents through enhancement of family‐based buffering processes (Brody et al. 2004). The program consists of six consecutive weekly sessions held in community facilities with separate, concurrent training sessions followed by joint parent–youth sessions during which families practice the skills learned in the separate sessions (Brody et al. 2012). Parenting sessions were designed to promote provision of instrumental support, emotional support and communication, problem‐focused coping, and occupational and educational mentoring. Youth sessions were designed to promote the development of goal setting and future orientation, to identify sources of support for goal attainment, and to cope with barriers and racial discrimination. The separate and concurrent sessions each lasted 1 hour resulting in a total of 12 hours of prevention training for each youth and parent.

### Data collection procedures

Demographic, parenting, and chronic anger variables were collected in the participants' home at baseline at youth age 17 (*M* = 17.7, SD = 0.72). Telomere length, chronic anger, and additional health variables, including body mass index (BMI) and blood pressure, were measured at a 5‐year follow‐up at age 22 (*M* = 22.02, SD = 0.98). Two African American field researchers visited the family's home and worked separately with the parent and youth. Interviews were conducted in a private location with no other family members present.

### Measures

#### Nonsupportive parenting

Nonsupportive parenting was measured through self‐report using measures assessing parent–child conflict and parental warmth and emotional support. Parent–child conflict was measured using parent report on two scales. The first, the Ineffective Arguing Inventory (IAI; Kurdek 1994), consisted of statements describing parent–child conflict with response scales ranging from 0 (*disagree strongly*) to 4 (*agree strongly*); *α *= 0.82. Examples include, “You and your child go for days being mad at each other” or “You and your child's arguments are left hanging and unsettled.” The second, the Arguing subscale from the Discussion Quality Scale (DQS, Brody et al. 1998), consisted of four statements regarding the frequency with which parents and youth argued over choice of friends, school or job, youth sex, and alcohol and other drugs, with response scales ranging from 1 (*never*) to 4 (*always*); *α *= 0.69 The IAI and DQS items were highly correlated (*r *= 0.51, *P *< 0.001) and were summed to form an indicator of parent–child conflict.

Parental warmth and emotional support was also measured using parental report on two scales. The first, the emotional support subscale from the Family Support Inventory (FSI; Wills et al. 1992), consisted of five items with parental rating scales ranging from 0 (*not at all true*) to 5 (*very true*); *α *= 0.79. Examples include “My child can trust me as someone to talk to,” and “When my child feels bad about something, I will listen.” The second, a revised version of the Emotional Support subscale from the Carver Support Scale (CSS; Carver et al. 1989), consisted of four items with parental rating scales ranging from 1 (*not at all true*) to 5 (*very true*); *α *= 0.77 Examples include, “My child discusses his/her feelings with me,” and “My child gets sympathy and understanding from me.” The CSS and FSI were highly correlated (*r *= 0.67, *P *< 0.001) and were summed to form an indicator of parental support. Parent–child conflict and parental warmth and support scores were standardized, and parental warmth and support was subtracted from parent–child conflict to create a measure of nonsupportive parenting. High values indicated high parent–child conflict and low levels of warmth and emotional support.

#### Chronic anger

Chronic anger was measured through youth self‐report using the anger subscale of the state‐trait anger expression inventory (Spielberger et al. 1983). The scale consists of 15 items describing feelings of anger over the past 3 months with youth rating scales ranging from 1(*always)* to 5 (*never*). Example items include, “I am furious” and “I feel angry”. Chronbach's alphas were 0.92 for baseline and 0.94 for follow‐up.

#### Genotyping

Youths' DNA was obtained using Oragene^™^ DNA kits (Genetek, Calgary, AB, Canada). Youths rinsed their mouths with tap water and deposited 4 ml of saliva in the Oragene sample vial. The vial was sealed, inverted, and shipped via courier to a central laboratory in Iowa City, where samples were prepared according to the manufacturer's specifications. Genotyping of *OXTR* rs53576 was determined for each youth using Applied Biosystems' TaqMan SNP Genotyping technology. Detailed information about extraction and genotyping procedures is available from the authors. Of the sample, 63.9% (*n *=* *122) were GG, 30.4% (*n *=* *58) were AG, and 5.7% (*n *=* *11) were AA. None of the alleles deviated from Hardy–Weinberg equilibrium, *χ*²(1) = 1.31, *P *= ns and no gender or intervention differences could be detected (*χ*² = 0.137; d.f. = 2, *P *=* *0.93 for gender; *χ*² = 2.156; d.f. = 2, *P *=* *0.34 for intervention status). Given the limited sample size of AA individuals, *OXTR* rs53576 alleles were grouped and coded as GG (1) versus AA/AG (0).

#### Telomere length

At the 5‐year follow‐up, certified phlebotomists visited the participant's home to draw a blood sample for telomere assessment. After the blood was drawn into serum separator tubes, it was frozen and delivered to the Psychiatric Genetics Lab at the University of Iowa for assaying. Telomere assessment of this sample has been described previously (Brody et al. 2015). Briefly, mononuclear (e.g., lymphocyte) cell pellets were generated using Ficoll separation (see Philibert et al. 2012) and the resulting lymphocyte cell pellets were prepared using a Qiagen QIAamp DNA Prep Kit according to the manufacturer's instructions. Telomere/standard (T/S) ratios were then calculated for each sample using a minor adaption of the improved quantitative polymerase chain reaction (PCR) method that (Cawthon 2009) developed, where DNA is amplified using a set of primers specific for either telomeric sequence or a single‐copy‐number standard gene (albumin). The ratios were subsequently normalized to a set of three internal LC DNA standards plated 8 times on each plate, resulting in a final telomere length range of −3.11 to 1.89 for this study.

#### Demographic and control variables

##### Intervention status and gender

Intervention condition was coded as randomization to intervention participation (1) or control condition (0). Gender was coded as male (1) and female (0).

##### Family socioeconomic status (SES) risk

SES risk has previously been associated with shorter telomere length outcomes (Price et al. 2013) and therefore was included in the study. Family SES risk was measured using six dichotomous variables that assessed presence or absence of the following characteristics: family poverty based on federal guidelines, primary caregiver unemployment, receipt of Temporary Assistance for Needy Families, primary caregiver single parenthood, primary caregiver education level less than high school graduation, and caregiver‐reported inadequacy of family income. The scores were summed to create the SES risk score. This technique has previously been used to forecast biomarkers of stress in African American adolescents (Brody et al. 2015).

##### Youth blood pressure and body mass index (BMI)

At the 5‐year follow‐up, resting blood pressure was monitored with a Critikon Dinamap Pro 100 (Critikon; Tampa, FL). Three readings were taken every 2 min, and the average of the last two readings was used. To create a single score, systolic and diastolic blood pressure scores were standardized and summed. Weight and height of each participant were then measured and used to calculate BMI (weight in kilograms divided by the square of height in meters) to control for the potential role of obesity in telomere length (Buxton et al. 2011).

### Statistical analysis

Chi‐square and *t*‐tests were used to compare study variables across the intervention and control conditions. To test the study hypotheses, three regression models were executed. The models testing gene by environment interactions followed the conventions of Aiken and West (Aiken and West 1991). Specifically, nonsupportive parenting was mean centered and interaction terms were calculated by creating a product term of the variables of interest (nonsupportive parenting, intervention condition and/or *OXTR* genotype). The first two models were intended to replicate the previous telomere findings in the subset of individuals with *OXTR* genetic data (see Brody et al. 2015). The first model tested the association between nonsupportive parenting at age 17 and telomere length at age 22. The second model tested the protective effects of the intervention program by incorporating an interaction term between nonsupportive parenting and intervention condition (PxI) on telomere length. The third model extended this further by including *OXTR* genetic status creating a three‐way interaction between nonsupportive parenting at age 17, intervention condition, and *OXTR* genetic status (PxIxG) on telomere length to test whether individuals with the GG genotype may show greater differences in telomere lengths across the nonsupportive and intervention conditions. All analyses controlled for the covariates of gender, family SES risk at age 17, BMI at age 22, and blood pressure at age 22. To further explore and visualize the direction of significant interactions, graphs were created using simple slope procedures. Estimate levels and slopes of telomere length outcomes for low (−1 SD) and high (+1 SD) nonsupportive parenting were graphed for each intervention and genotype group. Groups for PxI interaction include the intervention and control conditions. Groups for the PxIxG interaction include intervention GG genotype, intervention A+ genotype, control GG genotype, and control A+ genotype.

In the prior study, chronic anger was found to mediate the association between intervention participation and telomere length among those exposed to high levels of nonsupportive parenting. To extend this finding to our sample, we tested whether changes in anger from baseline to follow‐up mediate group differences in telomere length among youth who were exposed to high levels of nonsupportive parenting, as they were the most impacted by intervention or control placement, and those who had the *OXTR* GG genotype, as they were the ones most sensitive to the environment (*n* = 50, top 40% of nonsupportive parenting).

The hypothesis was tested using structural equation modeling with latent difference scores that reflect the degree to which anger changed from baseline to follow‐up (Fig. 2). First, regression coefficients were calculated for the association between intervention status and changes in anger (Path A) and the association between changes in anger and telomere length (Path B). Then, the indirect effect was quantified as the product of the two regression coefficients (A × B). In addition, nonparametric bootstrapping, which has been found to be sensitive in mediational analyses (Preacher and Hayes 2004), was used to obtain the bias‐corrected and accelerated confidence intervals (BCA) of the indirect effect for significance testing. The indirect, mediating effect was calculated 1000 times using random sampling with replacement to build a sampling distribution. Similar to the parent paper, gender, and family SES were controlled in the analysis. Chi‐square, t‐tests, and regression analyses were performed using SPSS 22 (RRID:rid_000042); the SEM model testing the mediation hypothesis was performed using Mplus 7.3.

## Results

### Descriptive statistics

More males were randomized to the intervention compared to control condition (*χ*²(1) = 6.76, *P *< 0.01), but the groups did not differ on any other study variables (*P *> 0.05). Descriptive statistics and correlations among study variables are presented in Table 1.

**Table 1:** Descriptive statistics and correlations among study variable (*N* = 191)

Variables	1	2	3	4	5	6	7	8
1. Gender, male	–							
2. Family socioeconomic risk (age 17)	0.017 *P* = 0.814	–						
3. Intervention, AIM	−0.188 *P* = 0.009*	−0.002 *P* = 0.980	–					
4. Nonsupportive parenting (age 17)	0.055 *P* = 0.454	0.017 *P* = 0.820	0.013 *P* = 0.853	–				
5. Telomere length (age 22)	−0.057 *P* = 0.436	0.029 *P* = 0.690	0.107 *P* = 0.141	−0.135 *P* = 0.062	–			
6. Body Mass Index (age 22)	−0.093 *P* = 0.201	−0.080 *P* = 0.271	0.098 *P* = 0.177	−0.002 *P* = 0.983	−0.080 *P* = 0.274	–		
7. Blood pressure (age 22)	0.417 *P* < 0.000**	0.063 *P* = 0.385	−0.080 *P* = 0.270	−0.015 *P* = 0.832	−0.036 *P* = 0.622	0.422 *P* < 0.000**	–	
8. rs53576 (A+ genotype vs. GG)	−0.022 *P* = 0.760	−0.032 *P* = 0.660	−0.041 *P* = 0.574	0.059 *P* = 0.421	−0.045 *P* = 0.533	−0.104 *P* = 0.152	−0.105 *P* = 0.150	–
Mean or *N*	73	1.94	0.52	0	−0.11	30.13	−0.02	122
SD or %	38%	1.21	0.50	3.05	0.76	10.03	1.88	64%

*P < 0.05; **P < 0.001.

SD, standard deviation.

### Nonsupportive parenting, intervention condition, and telomere length

**Figure 1:**
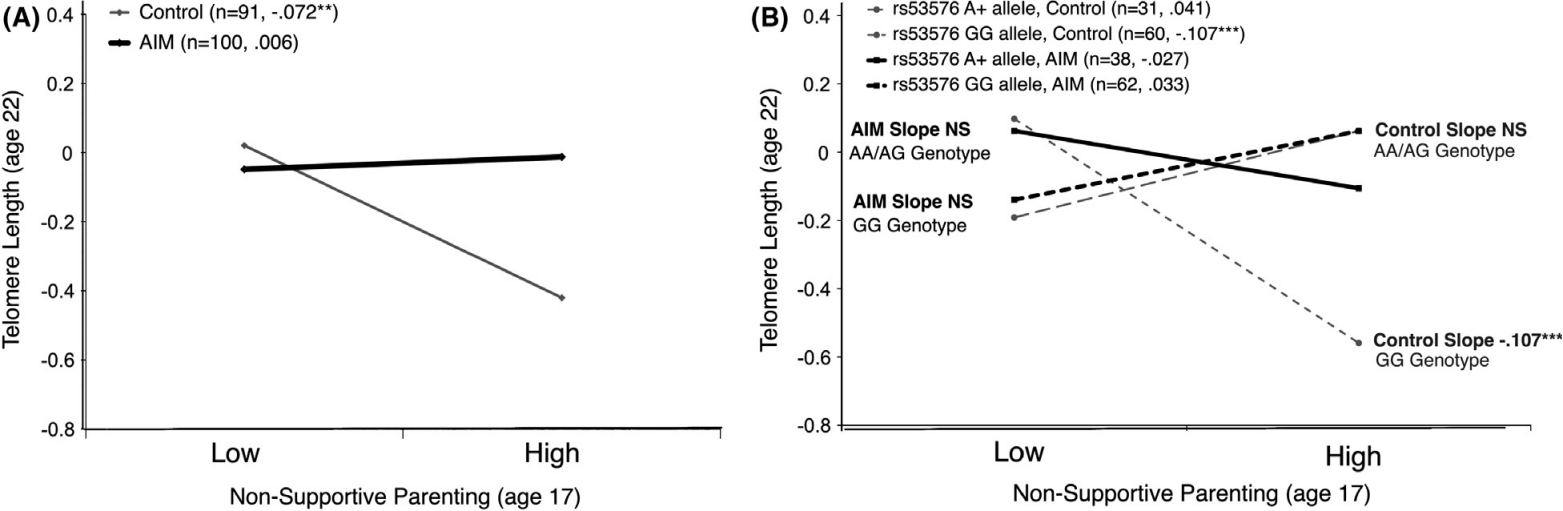
Nonsupportive parenting and telomere length outcomes by intervention and genotype status. Significant study interactions are depicted using simple slopes procedures for ± 1 standard deviation of nonsupportive parenting. Graphs display the association between nonsupportive parenting and telomere length outcomes across 5 years by a. intervention condition and b. intervention condition and *OXTR* rs53576 genotype. **P < 0.001. Rs53576 A+ allele, Control: *n* = 31; slope = 0.041; telomere length: Mean = −0.09, SD = 0.67, Range = −1.15 to −1.47. Rs53576 GG allele, Control: *n* = 60; slope = −0.107**; telomere length: Mean = −0.25, SD = 0.84, Range = −3.11 to 1.89. Rs53576 A+ allele, AIM: *n* = 38; slope = −0.027; telomere length: Mean = 0.04; SD = 0.72, Range = −1.58 to −0.04. Rs53576 GG allele, AIM: *n* = 62; slope = 0.033; telomere length: Mean = −0.03; SD = 0.74; Range = −2.16 to 1.58.

While the direct association between nonsupportive parenting and telomere outcomes was only significant at *P *= 0.07 (Table 2, Model 1; *β *= −0.131), an interaction emerged when taking intervention condition into account. Replicating previous findings, exposure to nonsupportive parenting at age 17 forecasted shorter telomere length at age 22, but this association was attenuated for those who participated in the AIM intervention (Table 2, Model 2; *β *= 0.223, *P *< 0.05). Among those exposed to high levels of nonsupportive parenting, only those randomized to the *control* condition evidenced significantly shorter telomeres at age 22 (Fig. 1A, simple slope = −0.072, *P *< 0.01), whereas those randomized to the intervention evidenced similar telomere lengths to those with more supportive, non at‐risk parenting scores at baseline (Fig. 1A, simple slope = 0.006, *P *= ns).

**Table 2:** Nonsupportive parenting, intervention status, and OXTR rs53576 as predictors of telomere length (*N* = 191)

Predictors	Telomere length (age 22)											
	Model 1				Model 2				Model 3			
	*B*	*SE*	*β*	*P*	*B*	*SE*	*β*	*P*	*B*	*SE*	*β*	*P*
1. Gender, male	−0.113	0.132	−0.073	0.392	−0.098	0.131	−0.063	0.455	−0.128	0.132	−0.082	0.333
2. Family socioeconomic risk (age 17)	0.013	0.041	0.023	0.758	0.011	0.040	0.019	0.790	−0.009	0.041	−0.017	0.818
3. Body Mass Index (age 22)	−0.007	0.006	−0.098	0.250	−0.009	0.006	−0.123	0.147	−0.011	0.006	−0.149	0.082
4. Blood pressure (age 22)	0.013	0.038	0.032	0.729	0.015	0.037	0.038	0.681	0.017	0.037	0.043	0.638
5. Nonsupportive parenting (age 17)	−0.032	0.018	−0.131	0.073	−0.072	0.025	−0.291	0.005^a^	0.041	0.051	0.167	0.421
6. Intervention, AIM	–	–	–	–	0.169	0.110	0.112	0.127	0.043	0.182	0.028	0.815
7. rs53576 (A+ vs. GG)	–	–	–	–	–	–	–	–	−0.166	0.167	−0.106	0.276
8. Parenting × AIM	–	–	–	–	0.078	0.036	0.223	0.030^a^	−0.069	0.063	−0.197	0.320
9. Parenting × rs53576	–	–	–	–	–	–	–	–	−0.149	0.060	−0.487	0.014^a^
10. AIM × rs53576	–	–	–	–	–	–	–	–	0.149	0.226	0.093	0.509
11. Parenting × AIM × rs53576	–	–	–	–	–	–	–	–	0.209	0.077	0.453	0.007^a^

SE, standard error.

Model 1: *F*(1, 185) = 3.257, *P *= 0.073, Δ*R* ^2^ = 0.017 (parenting main effect).

Model 2: *F*(1, 183) = 4.812, *P *= 0.030, Δ*R* ^2^ = 0.025 (parenting × AIM).

Model 3: *F*(1, 179) = 7.389, *P *= 0.007, Δ*R* ^2^ = 0.037 (parenting × AIM × rs53576).

a *P* < 0.05.

### Nonsupportive parenting, intervention condition, and OXTR genotype on telomere length

*OXTR* genetic status moderated the effects of nonsupportive parenting and intervention condition on telomere length across 5 years (Table 2, Model 3, *β *= 0.453, *P *< 0.01). Visualizing this three‐way interaction in Figure 1B, the association between parenting and telomere length is presented for low (−1 SD) and high (+1 SD) nonsupportive parenting for each genotype. *OXTR* rs53576 GG individuals, who are suggested to be more sensitive to the social environment, evidenced the shortest telomeres when exposed to nonsupportive parenting and randomized to the control condition (Fig. 1B, simple slope = −0.107, *P *< 0.001), and similar telomere lengths to the supportive parenting group when randomized to the intervention (Fig. 1B, simple slope = 0.033, *P *= ns). In contrast, AG/AA individuals showed no statistically significant difference in telomere lengths across all parenting and intervention conditions. In summary, the finding of protective effects of the AIM intervention on nonsupportive parenting and telomere length was found for the *OXTR* GG individuals, whereas those with the A allele showed similar telomere lengths across all conditions.

### Intervention participation, telomere length, and chronic anger

**Figure 2:**
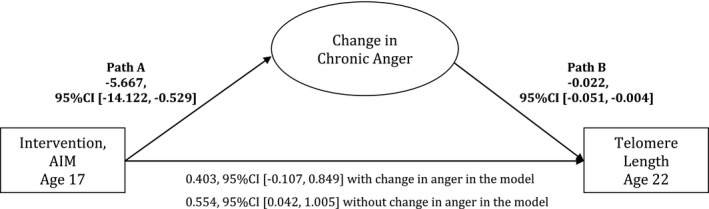
A mediational model of intervention status, change in chronic anger, and later telomere length. A mediational model of intervention status, change in anger from baseline to follow‐up, and telomere length at age 22 with socioeconomic‐related risk and gender controlled. Unstandardized coefficients are presented. Analyses were performed in the sample of participants with high nonsupportive parenting exposure at baseline and with the GG genotype, as they were most sensitive to the environments (*N* = 50). Indirect effect = 0.128, 95% CI [0.001, 0.470].

To follow the prior study, this study tested whether changes in chronic anger may mediate the association between intervention participation and telomere length outcomes given the occurrence of chronic anger following nonsupportive parenting and its link with stress response systems that may influence telomere shortening. This mediation model was tested among GG individuals in the top 40% of nonsupportive parenting (*n* = 50), as GG individuals were impacted by environmental condition and individuals exposed to nonsupportive parenting were most sensitive intervention placement. Results support the previous findings, whereby intervention status was significantly associated with changes in anger from baseline to follow‐up (*b* = −5.667, *P *< 0.05) and to telomere length (*b* = 0.554, *P *< 0.05), with GG individuals in the control condition reporting greater increases in chronic anger, and this associating with shorter telomere length (Fig. 2). In comparison, GG individuals in the intervention condition had less of an increase in chronic anger, which was then associated with longer telomeres. The significant negative coefficient between changes in anger and telomere length (*b* = −0.022, *P *< 0.05) indicated that, the more a participant's anger increased from baseline to follow‐up, the shorter his or her telomere length was at age 22. Changes in anger significantly mediated the intervention effect on telomere length, such that the intervention effect was significantly reduced after accounting for changes in anger (indirect effect estimates = 0.128, BCA = 0.001–0.470 with 1000 bootstrapping), which reduced the intervention effect to a nonsignificant relation (Fig. 2).

## Discussion

This study identified a significant three‐way interaction whereby *OXTR* genetic status moderated the association between nonsupportive parenting and exposure to a family‐based prevention intervention program on long‐term telomere length outcomes. Adolescent youth with the *OXTR* GG genotype were the most sensitive to both the parental support and intervention conditions, evidencing the shortest telomeres when exposed to high levels of nonsupportive parenting (high parent–child conflict and low levels of warmth and emotional support), yet longer telomeres when exposed to these conditions but randomized to participate in the intervention. In contrast, youth with AA/AG genotypes showed no statistical difference in telomere lengths across all parental support and intervention conditions. This study also supports the potential role of chronic anger in mediating these findings, whereby GG individuals exposed to high nonsupportive parenting and randomized to the control had a greater increase in chronic anger, compared to those randomized to the intervention, and this increase associated with shorter telomere lengths.

These findings can be interpreted in the context of genetic sensitivity and the Social Sensitivity hypothesis for oxytocin (Bartz et al. 2011). According to this hypothesis, oxytocin functions to increase the salience of social cues and thus influences an individual's sensitivity to the social environment, both positive and negative. Applied to the *OXTR* genetics literature, the rs53576 G allele has been conceptualized as conferring increased social sensitivity (Bartz et al. 2011). This study further supports this interpretation and extends the *OXTR* literature by suggesting that differential phenotypic outcomes by environmental context may be present at the level of telomere length outcomes. In this study, GG individuals evidenced greater variation in telomere length outcomes in association with environmental context. This finding suggests that the adverse social aspects of high conflict and low warmth parent–child environments, and the positive aspects of intervention participation and youth and parental engagement in socioemotional and instrumental support development may be particularly relevant and impactful for these individuals.

One mechanism by which the intervention may associate with telomere length outcomes is through an impact on chronic anger, as explored in the prior paper. Supporting the prior findings, we found that GG individuals exposed to high levels of nonsupportive parenting and randomized to the control condition had a significant increase in chronic anger ratings over 5 years, and this increase was associated with greater telomere shortening. In comparison, GG individuals randomized to the intervention had less of an increase in chronic anger, and longer telomeres. GG individuals exposed to high levels of social conflict have previously been found to exhibit higher levels of adverse behavioral and emotional outcomes , including emotion dysregulation (Bradley et al. 2011) and higher levels of conduct disorder behaviors (Smearman et al. 2015), supporting these findings. Furthermore, chronic anger may underlie the association between intervention participation and telomere length as chronically angry states are suggested to result in more frequent activation of stress response systems thought to contribute to telomere shortening, such as frequent activation of the sympathetic nervous system and the hypothalamic‐pituitary‐adrenal axis (Blackburn 2005; Aviv 2008).

While intriguing, there are some important points to consider when interpreting the study findings. First, the biological function of allelic variation in rs53576, or of genotypes in linkage disequilibrium, is still not known. Rs53576's presence in the large 3rd intron of the gene may point to potential influence on gene regulation (Mizumoto et al. 1997; Gimpl and Fahrenholz 2001) but research exploring mechanistic questions is needed. Following this, grouping of the heterozygote AG individuals has primarily been influenced by sample size limitations, with the majority of studies grouping AG with AA individuals due to a limited sample size of AA genotypes. While A‐dominant (AG/AA) grouping can facilitate appropriate statistical testing and social sensitivity has been reported for both G‐dominant (Bradley et al. 2011, 2013) and A‐dominant (Sturge‐Apple et al. 2012) grouping, it is plausible that grouping may influence study outcomes (Smearman et al. 2015) and supports further exploration of allelic grouping and underlying biological mechanisms.

According to the Social Sensitivity hypothesis, it may be expected that those with the GG genotype would evidence the longest telomeres when exposed to highly supportive parenting and intervention participation, above those of the AA/AG genotype. However, telomere lengths across these groups were not statistically different. This may be due to limitations of the study measures or current sample. The parenting scales may have not been as sensitive on the higher end of positive parenting; however, while conflict was primarily focused on adverse interactions, warmth and emotional support focused on more positive interactions. Sample size is an important consideration for genetic interaction studies, as discussed in detail below, and it is also possible that the differences may become apparent in a larger sample. However, it is plausible that this finding is representative of the sample. This sample is drawn from a rural community of primarily working poor who may be experiencing additional sources of stress that could limit the upper end of telomere length outcomes. While SES risk was included in the study, those with the GG genotype may be influenced by other potential sources of stress even if parenting‐based stressors are not present or are addressed through the intervention. Finally, it is also possible that those with the GG genotype may be more impacted by negative social environments on telomere length outcomes than positive environments, with telomere lengths in positive environments resembling lengths of those with the A allele. These questions could be addressed through additional research and thorough assessments of social stress and positive social environments on long‐term telomere length outcomes across *OXTR* genotypes.

This study is not without limitations. The study was conducted with an African American sample of relatively low SES. Therefore, these results may have limited generalizability. However, few studies have utilized African Americans, particularly African American youth, in assessments of telomere length outcomes (Mitchell et al. 2014) and *OXTR* genetic moderation on phenotypic outcomes (adult samples: Bradley et al. 2011, 2013). Therefore, the study provides an important addition to the literature by utilizing this population.

As with all gene–environment studies, sample size limitations are an important consideration. The current sample size is relatively small, highlighting concern for spurious findings (Duncan and Keller 2011). Therefore, replication is warranted. Considering this, there are some unique aspects to utilizing this sample for an interaction analysis. Intervention programs allow for direct modification of the environment in a controlled setting, allowing researchers to more directly assess the relationship between varying environments and phenotypic outcomes (Rutter 2005; Brody et al. 2013b, 2014). This direct modification provides a more controlled assessment of these relationships, as well as whether the relationships may be moderated by genetic status. Added to this, the current sample provides opportunity for exploration of both negative and intentionally more positive environments through nonsupportive parenting assessments and direct implementation of a program intended to promote positive relationship development and family‐based buffering processes. Future research drawing from larger samples will allow for replication and further exploration of these findings.

Finally, telomere assessment was collected at 5 years post intervention, in response to the growing literature in this field. While informative, this collection timing is limited and future research utilizing both pre and post telomere assessments should be conducted to more directly test questions of telomere length change over time, as well as the potential for genetic moderation of this change.

## Conclusions

The telomere literature has continued to grow with numerous studies to date reporting associations between exposure to chronic stress and shorter telomere length outcomes (Price et al. 2013). In addition to these negative environments, positive social environments, such as healthy social ties (Uchino et al. 2012), and greater perceived social support (Carroll et al. 2013), have now repeatedly been associated with longer overall telomere lengths. Our research group previously reported that implementation of a prevention program aimed at promoting positive parent–child interactions and family‐based stress‐buffering processes may result in greater overall telomere lengths among those originally exposed to stressful, nonsupportive parenting environments (Brody et al. 2015). Extending this into the growing literature of genetic sensitivity to environmental contexts, this study found that *OXTR* genetic variation influenced responsiveness to environmental contexts at this biological level. As hypothesized, individuals with the rs53576 GG genotype evidenced greater variability, showing the shortest telomere lengths across 5‐years when exposed to nonsupportive parenting and randomized to the control condition, yet similar telomere lengths to non at‐risk groups when randomized to the intervention. In contrast, those with the A allele showed no statistical difference in telomere lengths across all parental support and intervention conditions. These findings highlight the role of prevention in attenuating the impact of stressful exposures yet the potential importance of individual differences in genetic sensitivity to these contexts.

## Conflict of Interest

The authors have no conflicts of interest.
